# Proteomic analysis of diabetic retinas

**DOI:** 10.3389/fendo.2023.1229089

**Published:** 2023-08-25

**Authors:** Christopher R. Starr, Assylbek Zhylkibayev, James A. Mobley, Marina S. Gorbatyuk

**Affiliations:** ^1^Department of Optometry and Vision Science, University of Alabama at Birmingham, Birmingham, AL, United States; ^2^Department of Anesthesiology and Perioperative Medicine, University of Alabama at Birmingham, Birmingham, AL, United States

**Keywords:** diabetes, retina, proteomics, diabetic retinopathy, glycosylation

## Abstract

**Introduction:**

As a metabolic disease, diabetes often leads to health complications such as heart failure, nephropathy, neurological disorders, and vision loss. Diabetic retinopathy (DR) affects as many as 100 million people worldwide. The mechanism of DR is complex and known to impact both neural and vascular components in the retina. While recent advances in the field have identified major cellular signaling contributing to DR pathogenesis, little has been reported on the protein post-translational modifications (PTM) - known to define protein localization, function, and activity - in the diabetic retina overall. Protein glycosylation is the enzymatic addition of carbohydrates to proteins, which can influence many protein attributes including folding, stability, function, and subcellular localization. *O*-linked glycosylation is the addition of sugars to an oxygen atom in amino acids with a free oxygen atom in their side chain (i.e., threonine, serine). To date, more than 100 congenital disorders of glycosylation have been described. However, no studies have identified the retinal *O*-linked glycoproteome in health or disease. With a critical need to expedite the discovery of PTMomics in diabetic retinas, we identified both global changes in protein levels and the retinal *O*-glycoproteome of control and diabetic mice.

**Methods:**

We used liquid chromatography/mass spectrometry-based proteomics and high throughput screening to identify proteins differentially expressed and proteins differentially *O*-glycosylated in the retinas of wildtype and diabetic mice.

**Results:**

Changes in both global expression levels of proteins and proteins differentially glycosylated in the retinas of wild-type and diabetic mice have been identified. We provide evidence that diabetes shifts both global expression levels and *O*-glycosylation of metabolic and synaptic proteins in the retina.

**Discussion:**

Here we report changes in the retinal proteome of diabetic mice. We highlight alterations in global proteins involved in metabolic processes, maintaining cellular structure, trafficking, and neuronal processes. We then showed changes in *O*-linked glycosylation of individual proteins in the diabetic retina.

## Introduction

Diabetes is a metabolic disorder associated with the development of hyperglycemia due to a high blood glucose level. The global diabetes prevalence in 2019 was estimated to be 463 million people, rising to 578 million by 2030 and 700 million by 2045 (1). Type-1 (T1D) and Type-2 diabetes (T2D) differ in their pathogenesis. T1D, or juvenile diabetes, is autoimmune in nature and results in the pancreas producing very little insulin. T2D patients produce insulin but have tissues that are resistant to the effects of insulin. Diabetic retinopathy (DR) is a life-limiting complication of diabetes, a disease estimated to affect 100 million people worldwide and is associated with neuromicrovascular dysfunction (2). DR pathogenesis is characterized as nonproliferative (NPDR) -or early stage, and advanced proliferative (PDR) (reviewed in (3)]. Retinas of patients with NPDR may show clear clinical signs such as microaneurysms, hemorrhages and intraretinal microvascular abnormalities. Patients with PDR develop pathological preretinal neovascularization. Neurodegeneration and retinal vascular dysfunction are hallmarks of DR, with neovascularization being the primary clinical focus. However, recent research projects have identified various neuronal abnormalities present in models of DR. These include cone dysfunction and thinning of the retinal ganglion cell (RGC) layer (4, 5). Diabetes is a systemic disease with multiple features having the ability to impact the retina including hyperglycemia, advanced glycation end-products (AGE) adduct accumulation, dyslipidemia, and hypertension. Hyperglycemia alone accounts for 10% of the risk factor for developing DR (6). Abnormal glucose flux and hyperglycemia are believed to activate protein kinase C signaling, hexose monophosphate shunt, and AGE, which demonstrates the incredibly complex molecular mechanisms of DR pathogenesis (7). Unfortunately, the identification of relevant treatments for DR has been delayed by the inaccessibility of suitable animal models (8–10).

Recent breakthroughs in protein purification strategies and current proteomic technologies (11) make it possible to identify the proteomics of healthy and diseased retinas. Despite these advances, the research field is significantly lagging when it comes to identifying proteins modified in diseased retinas, despite knowledge of the changes in protein regulation in the retina being critical for drug development (12).

Protein modifications that occur either co-translationally or posttranslationally (post-translational modifications or PTMs) constitute a key step in protein biosynthesis and in the regulation of protein function, activity, and localization. Phosphorylation can, for example, dynamically alter enzymatic activity. In addition, methylation and acetylation, specifically of histones, can regulate gene expression. Protein ubiquitination can vastly impact protein function or even signal for its degradation through the ubiquitin proteosome system.

Protein glycosylation is the enzymatic addition of carbohydrate chains to proteins, which greatly influences protein folding, stability, function, and subcellular localization [reviewed in (13–16)]. Glycosylation is dependent on the cytosolic synthesis of nucleotide-sugars, with the exception of CMP-NeuAc being synthesized in the nucleus, though N-linked glycosylation is initiated in the ER, co-translationally while *O*-glycosylation is initiated in the Golgi. (17). Glycans are assembled and added to proteins for further modifications. N-linked glycosylation involves the enzymatic linkage of glycans to proteins, begins in the ER, continues in the Golgi, and greatly influences protein stability and function. As compared to N-linked glycosylation, *O*-linked glycosylation occurs on the side chain of serine or threonine instead of asparagine residues. *O*-linked glycans are incredibly diverse as they are initiated by a plethora of different monosaccharides that yields even higher order diversity when forming *O*-glycans (18). For example, the most common *O*-linked glycosylation, *O*-GalNAc, is initiated in the Golgi and has significant influence on the cellular localization, binding partners, and function of proteins. *O*-Linked β-N-acetylglucosamine (*O*-GlcNAcylation) occurs in the cytoplasm and nucleus. *O*-glycosylation is one of the most abundant PTMs; however, detailed analyses of this PTM has been historically limited due to the shortage of appropriate methods.

Studies of glycosylation disorders have led to the discovery of tissue specific glycosylation pathways [reviewed in (15)]. In fact, more than 100 disorders of glycosylation have been described to date. Even with clear evidence that altered glycosylation can lead to disease, the functional glycoproteome remains poorly studied in both healthy and diseased tissue. Furthermore, no extensive studies have identified the retinal *O*-linked glycoproteome in health or disease, although a critical need of discovery glycoproteomics exists in the field. A few exceptions have been presented by published work of Gurel and Sheibani, (19) Xing et al., (20), and Lei et al. (21) which have highlighted the importance of *O*-glycosylation in diabetic retinas in general and have not identified *O*-glycosylation of individual proteins. In the present study, we identify *O*-glycosylation protein pattern in healthy and diseased retinas with nano high-performance liquid chromatography (HPLC)/mass spectrometry (MS) and detected differential retinal global protein expression in the streptozotocin (STZ)-induced diabetes mouse model. Though, we report evidence of robust changes in multiple cellular pathways, in this study, we primarily focus on the changes in metabolic and synaptic proteins at both the protein and *O*-glycosylation levels.

## Results

### Discovery proteomics in the retinas of diabetic mice

**Figure 1:**
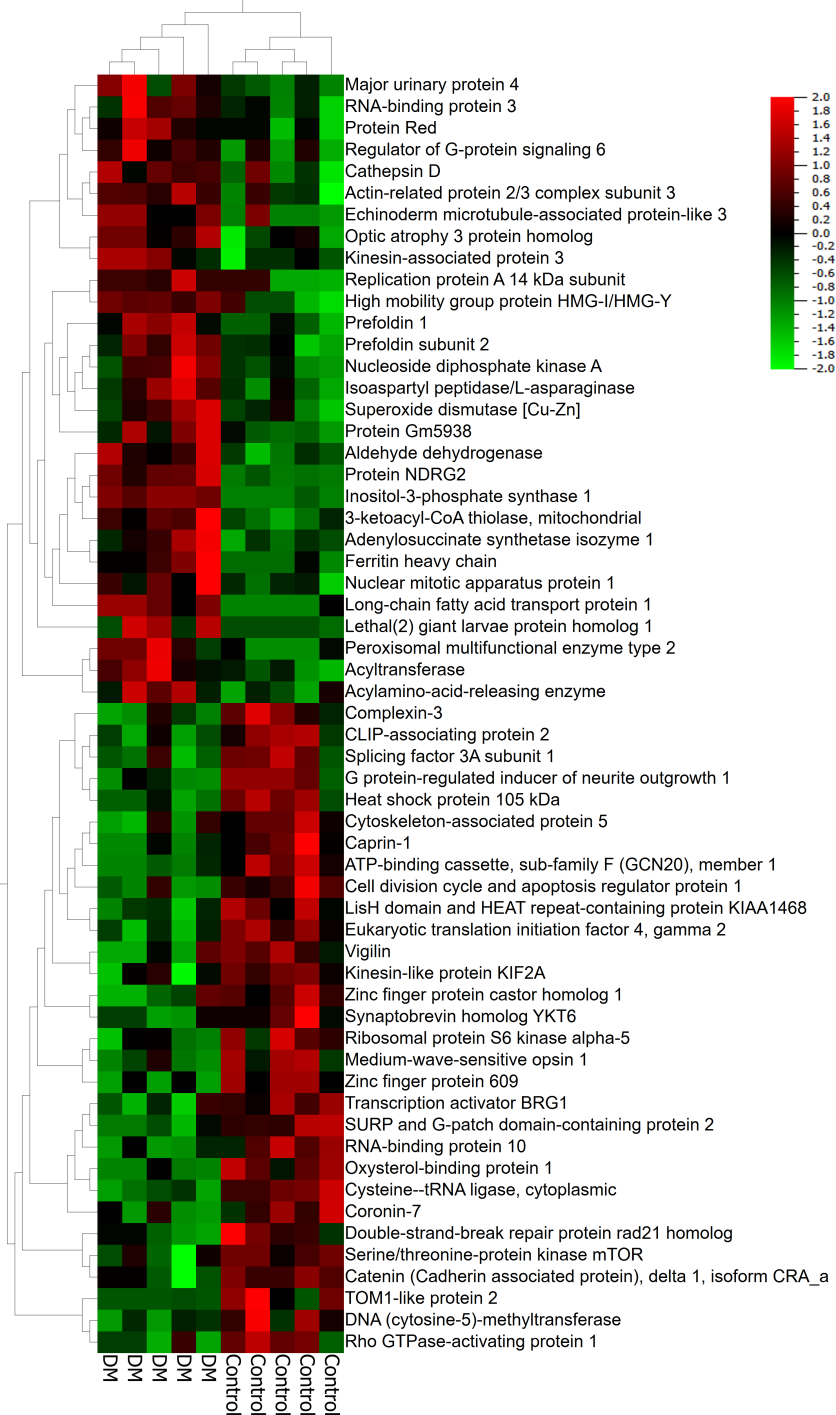
A heatmap of major proteins modified in the diabetic retina. Increased and decreased proteins are indicated in red and green.

To facilitate discovery proteomics of diabetic retinopathy, we utilized a well characterized mouse model of diabetes, STZ-induced diabetes (3, 22–25) using a non-biased proteomics approach. Proteins were extracted from retinal tissue of diabetic mice, trypsin digested and analyzed using nano-HPLC/MS at 12 weeks post injection. We first identified differences in global proteomics between retinas from control and STZ mice (**Figure 1**, **Tables 1**, **2**, and **Supplementary Table S1**). In this dataset, we identified significant changes in peptides corresponding to 145 proteins. **Figure 1** shows a heatmap of global protein changes in diabetic retinas. **Table 1** highlights global top hits, 30 proteins of elevated abundance and 30 proteins with reduced abundance— and indicates their fold changes and *p-*values.

**Table 1:** The top 30 increased and decreased differentially expressed proteins in the diabetic retinas.

Top 30 increased proteins	GeneID	Accession#	Fold increase	P value	Top 30 decreased proteins	GeneID	Accession#	Fold decrease	P value
Protein Lcn11	227630	A2BHR2	13.34	0.014	Caprin-1	53872	Q60865	0.53	0.013
Secretoglobin family 2B member 24	233090	Q7M747	12.73	0.038	DNA (cytosine-5)-methyltransferase	13433	Q7TSJ0	0.49	0.022
ABC transporter A subfamily member, A8a	217258	A4PBQ7	12.48	0.008	Synaptobrevin homolog YKT6	56418	Q9CQW1	0.49	0.032
Inositol-3-phosphate synthase 1	71780	Q9JHU9	6.97	4E-8	ATP-binding cassette, sub-family F (GCN20), member 1	224742	Q5RL55	0.46	0.001
60S ribosomal protein L28	19943	P41105	6.82	0.023	Arf-GAP with GTPase, ANK repeat and PH domain-containing protein 1	347722	Q8BXK8	0.43	0.034
Protein LEG1 homolog	67719	Q8C6C9	6.68	0.000	SURP and G-patch domain-containing protein 2	234373	Q8CH09	0.42	0.001
Eukaryotic translation elongation factor 1 epsilon-1	66143	Q9D1M4	5.77	0.019	Pre-mRNA-processing factor 40 homolog A	56194	Q9R1C7	0.40	0.039
Ribosomal protein S23	66475	Q497E1	4.18	0.037	Leucyl-cystinyl aminopeptidase	240028	Q8C129	0.39	0.024
Eukaryotic translation initiation factor 5	217869	P59325	3.78	0.017	Cysteine--tRNA ligase, cytoplasmic	27267	Q9ER72	0.39	0.000
Emopamil-binding protein-like	68177	Q9D0P0	3.66	0.018	Active breakpoint cluster region-related protein	109934	Q5SSL4	0.38	0.035
Frataxin, mitochondrial	14297	O35943	3.65	0.018	Probable global transcription activator SNF2L2	67155	Q6DIC0	0.37	0.031
Ubiquitin-conjugating enzyme E2 D2B	73318	Q6ZWY6	3.45	0.025	Coronin-7	78885	Q9D2V7	0.37	0.012
Peroxisomal multifunctional enzyme type 2	15448	P51660	3.41	0.007	Ribosomal protein S6 kinase alpha-5	73086	Q8C050	0.36	0.007
Major urinary protein 4	17843	P11590	3.26	0.015	26S proteasome non-ATPase regulatory subunit 9	67151	Q9CR00	0.35	0.044
Stomatin-like protein 2, mitochondrial	66592	Q99JB2	3.18	0.020	Ataxin-2-like protein	233871	Q7TQH0	0.34	0.024
Glycoprotein m6b, isoform CRAf	14758	Q3US81	3.13	0.013	RNA-binding protein 10	236732	Q99KG3	0.33	0.002
Rps16 protein	20055	Q5CZY9	2.86	0.045	Signal transducer and activator of transcription 5B	20851	P42232	0.32	0.027
BolA-like protein 2	66162	Q8BGS2	2.70	0.037	Death-inducer obliterator 1	23856	Q8C9B9	0.31	0.009
ATP-binding cassette sub-family A member 8-B	27404	Q8K440	2.69	0.040	Tubulin--tyrosine ligase-like protein 12	223723	Q3UDE2	0.28	0.022
Protein Gm5938	546335	A2AEN9	2.65	0.012	Oxysterol-binding protein 1	76303	Q3B7Z2	0.27	0.000
Nucleoside diphosphate kinase B	18103	Q01768	2.53	0.016	G protein-regulated inducer of neurite outgrowth 1	26913	Q3UNH4	0.24	0.003
Optic atrophy 3 protein homolog	403187	Q505D7	2.51	0.006	SLIT-ROBO Rho GTPase-activating protein 2	14270	Q91Z67	0.22	0.014
Prefoldin 1	67199	Q9CQF7	2.20	0.005	Protein LZIC	69151	Q8K3C3	0.19	0.004
3-ketoacyl-CoA thiolase, mitochondrial	52538	Q8BWT1	2.12	0.004	Protein Rasa1	218397	E9PYG6	0.18	0.013
Rab5B	19344	Q0PD56	2.09	0.049	General transcription factor IIIC, polypeptide 3	98488	Q3TMP1	0.16	0.038
ADP-ribosylation factor-like protein 8A	68724	Q8VEH3	2.05	0.046	High mobility group protein B3	15354	O54879	0.14	0.003
Mitochondrial pyruvate carrier 1	55951	P63030	2.00	0.044	Trafficking protein particle complex subunit 12	217449	Q8K2L8	0.14	0.006
Acylamino-acid-releasing enzyme	235606	Q8R146	1.99	0.027	Rho GTPase-activating protein 44	216831	Q5SSM3	0.09	0.001
Isoaspartyl peptidase/L-asparaginase	66514	Q8C0M9	1.98	0.014	Jumonji domain containing 1B	277250	B9EKS2	0.09	0.022
Kinesin-associated protein 3	16579	P70188	1.98	0.020	Alpha-1-antitrypsin 1-5	20704	Q00898	0.04	0.023

**Table 2:** The protein exclusively expressed either in the diabetic or normal retina.

Proteins	GeneID	Accession #	Present/Undetectable
Liprin-alpha 2	327814	B8QI34	Undetectable
Melanoma inhibitory activity protein 3	338366	Q8BI84	Undetectable
Phosphatidylinositol-3,4, 5-trisphosphate-dependent Rac exchange factor 1	277360	B9EKR4	Undetectable
Phosphomevalonate kinase	68603	Q9D1G2	Undetectable
Vacuolar protein sorting-associated protein 16 homolog	80743	Q920Q4	Undetectable
Prolyl-tRNA synthetase-associated domain-containing protein 1	67939	Q9D820	Present

Because diabetes is a metabolic disorder that alters metabolic pathway in the retina leading to neurodegeneration, in this manuscript, we primarily focus on the results of global protein changes in retinal metabolism and neuronal processes.

#### Global changes in metabolic pathways in diabetic retinas

Upon analyzing the datasets, it became clear that many of the top hits are involved in one or more metabolic processes. For instance, diabetic mice had elevated levels of inositol-3-phosphate synthase 1 (Isyna1), Nucleoside diphosphate kinase A (Nme1), 3-ketoacyl-CoA-thiolase- mitochondrial (Hadhb), Aldehyde dehydrogenase, Isoaspartyl peptidase/L-asparaginase (Asrgl1), Adenylosuccinate synthetease isozyme 1 (AdSS1), Inositol-3-phosphate synthase 1(Isyna1), long-chain fatty acid transport protein 1(Fatp1), and Acylamino-acid-releasing enzyme (Acph) suggesting that multiple metabolic pathways could be altered in diabetic retinas. Protein Red, high mobility group protein HMG-I/HMG-Y and RNA binding protein 3 — proteins involved in RNA processing, were also elevated in diabetic retinas. Anti-oxidant superoxide dismutase Sod1 was also elevated in diabetic retina (**Figure 1**). In addition, levels of proteins involved in protein processing and folding, Prefoldin 1 and Prefoldin subunit 2, were also increased. Taken together, these elevated proteins indicate an increase of components involved in a significant number of metabolic processes in diabetic retinas.

**Figure 2:**
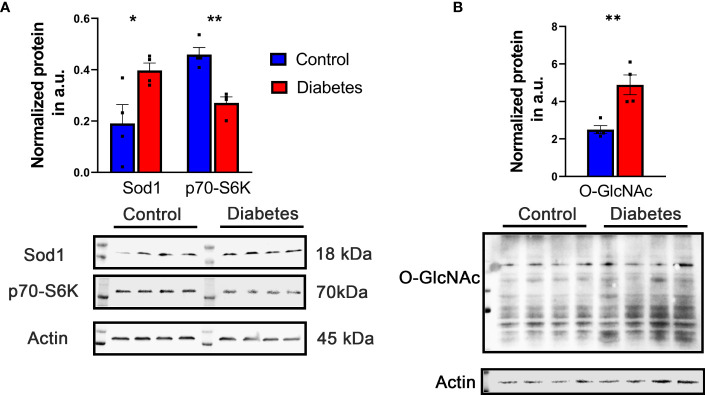
Representative increased and decreased proteins were detected by western blot. **(A)** Increase in Sod1 and decrease in S6K level were detected in diabetic retina validating results obtained with LC-MS -based approach. **(B)** The level of *O*-GlcNAc proteins detected with CDT110.6 antibody was enhanced in diabetic retina. *p<0.05 , **p<0.01.

Certain cellular processes appeared to be globally reduced in the retinas of diabetic mice. Of note, a number of proteins involved in metabolic processes were reduced in diabetic retinas. Some of the top hits reduced in diabetic retinas include Vigilin, Splicing factor 3A subunit 1 (Sf3a1), ATP-binding cassette sub-family F member 1 (Abcf1), SURP and G-patch domain-containing protein 2 (Sugp2), Eukaryotic translation initiation factor 4 gamma 2 (Eif4g2), Ribosomal protein S6 kinase alpha-5 (Rps6ka5), RNA-binding protein 10 (Rbm10), Serine/threonine-protein kinase mTOR (**Figures 1**, **2A** and **Table 1**).

**Figure 3:**
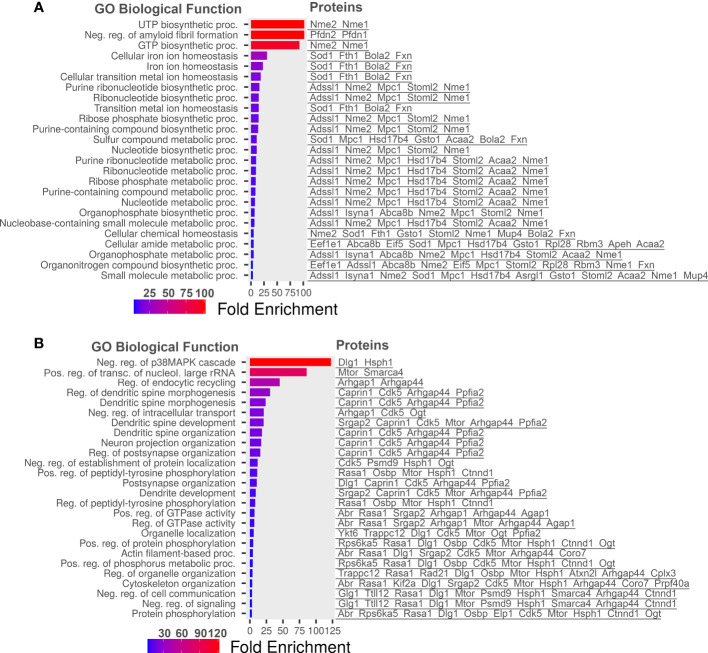
Go Biological processes for proteins significantly changed in our dataset. **(A)** Biological processes of proteins increased in diabetic retinas. **(B)** Go Biological processes of proteins reduced in diabetic retinas. Left is list of GO Biological Processes and right is list of proteins related to the biological process. Graphs and lists generated with ShinyGO software.

Statistically significant increased and decreased proteins in diabetic retinas were then plotted into ShinyGo program to group them by Gene Ontology (GO) biological processes using a 1.5 threshold for increased and 30% for decreased proteins (**Figure 3**). **Figure 3** depicts the most abundant biological process occurring in diabetic retinas using the fold enrichment criterion. The negative regulation of p38K cascade was one of the drastically decreased biological processes in the diabetic retinas.

**Figure 4:**
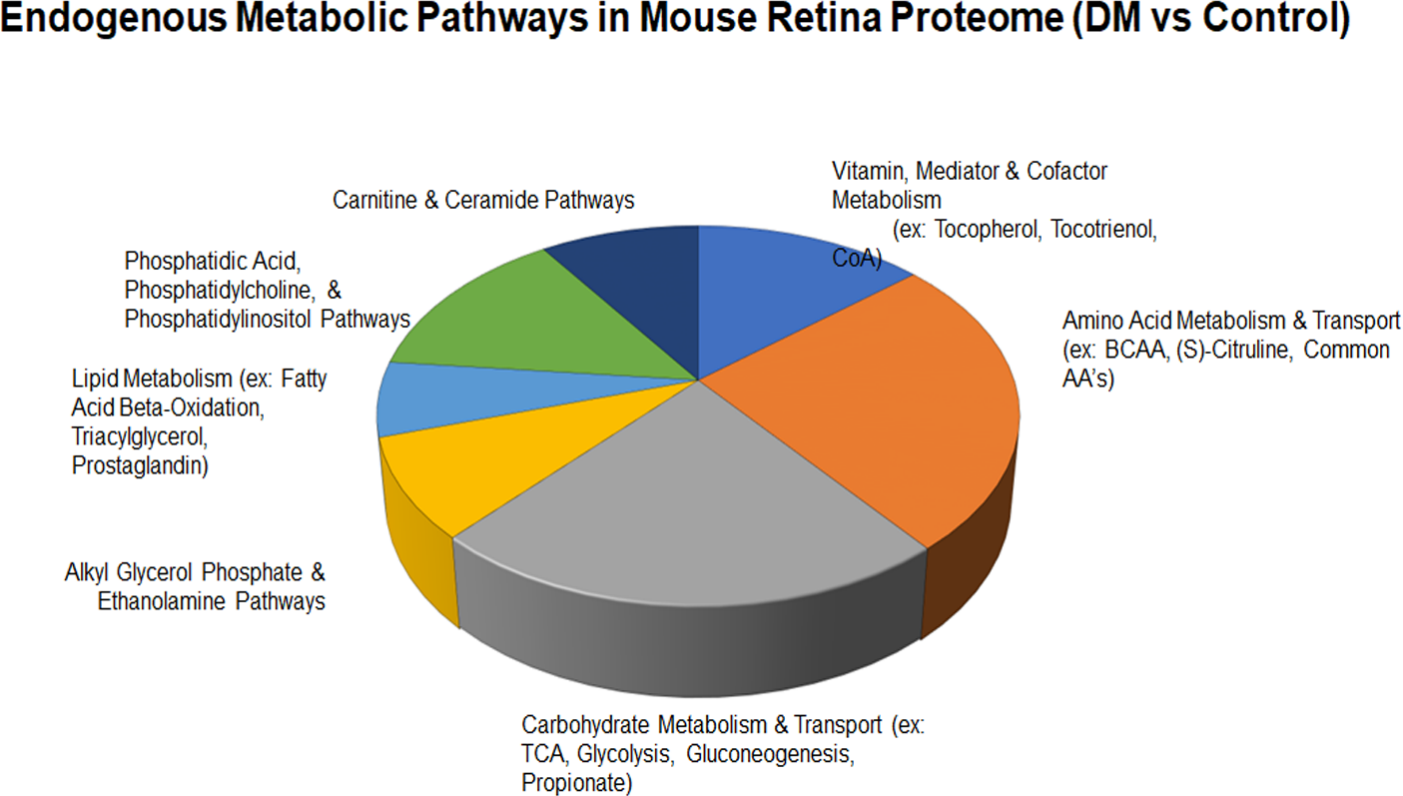
The major cellular pathways altered in diabetic retina are shown. Carbohydrate and amino acid metabolism presented almost 50% of all endogenous metabolic pathways modified in the diabetic mouse retina. The alteration in vitamin metabolism, carnitine & ceramide pathways, and phosphatidic acid pathways accounted about 35%. The remaining 15% of metabolism altered in the diabetic retinas was associated with lipid metabolism and alkyl glycerol phosphate and ethanolamine homeostasis.

**Figure 4** highlights global changes in metabolic processes in more depth. All significant changes in global proteomics can be found in **Supplementary Table S1**. **Table 2** depicts proteins either exclusively identified or undetectable in the diabetic retinas. For example, we learned that VPS16 that plays an important role in segregation of intracellular molecules into distinct organelles was undetectable in diabetic retinas. The VPS16 is known to be predominantly associated with late endosomes/lysosomes, and therefore, may mediate vesicle trafficking steps in the endosome/lysosome pathway in diabetic retinas. Moreover, loss of function of VPS16 gene causes early onset dystonia associated with lysosomal abnormalities (26). Another example is PRXD1 present exclusively in diabetic retinas. We also noted significant changes in levels of many proteins involved in metabolic processes (**Figure 4**). The major changes occurred in the expression of proteins responsible for lipid metabolism: fatty acid β-oxidation (3-fold), phosphatidylcholine pathway (2.7-fold), triacylglycerol pathway (2.6-fold), ceramide pathway (2.3-fold), and carbohydrate metabolism: propionate pathway and transport (2-fold). **Figures 1**, **2**, **4** depict just a fraction of the proteins and cellular processes altered in our dataset, for a complete list of all proteins, cellular processes, functions, and more, see **dataset 1 and Supplementary File 1**.

#### Global shift in levels of proteins involved in cellular structure, trafficking and neuronal processes

Various proteins involved in structural integrity, cellular trafficking and/or synaptic function were also reduced in diabetic mice. For example, CLIP-associating protein 2 (Clasp2), Complexin-3, G protein-regulated inducer of neurite outgrowth 1 (Grin1), Cytoskeleton-associated protein 5 (Ckap5), Caprin-1, Synaptobrevin homolog YKT6, Kinesin-like protein KIF2A, Coronin-7, Catenin delta-1, and TOM1-like protein 2 (Tom1l2) were significantly diminished. Considering the highlighted above changes in levels of proteins responsible for metabolic signaling and of those involved in structural, neuronal processes and trafficking, we theorize that the retinal neurons in diabetic mice are altering their metabolism and neuronal processes to shift the energy expenditure towards molecular mechanisms responsible for the life support of the somas.

### Identifying the retinal *O*-glycoproteome in diabetic mice

Protein glycosylation is the enzymatic addition of carbohydrates to proteins. Glycosylation can influence protein folding, stability, function, and localization. There are diseases associated with faulty glycosylation machinery as well as changes in glycosylation of key proteins. For example, defect in *O*-linked glycosylation causes familial tumoral calcinosis, severe autosomal recessive metabolic disorders showing massive calcium deposits in the skin and subcutaneous tissues (27). The disorder is due to mutations in *GALNT3*, one of the *O*-GalNAc transferases in mucin *O*-glycosylation. Another example is Tn-syndrome, which is caused by somatic mutations in the X-linked gene *COSMC* encoding a highly specific chaperone required for the proper folding and normal activity of β1-3 galactosyltransferase needed for the synthesis of core 1 *O*-glycans (28). Though studies on retinal diseases have highlighted proteins whose glycosylation is vital for their functions, to date, no large-scale datasets pertaining to *O*-glycosylation in the retina have been published. Therefore, we next sought to elucidate how the retinal *O*-glycoproteome was altered in diabetic retinas.

**Figure 5:**
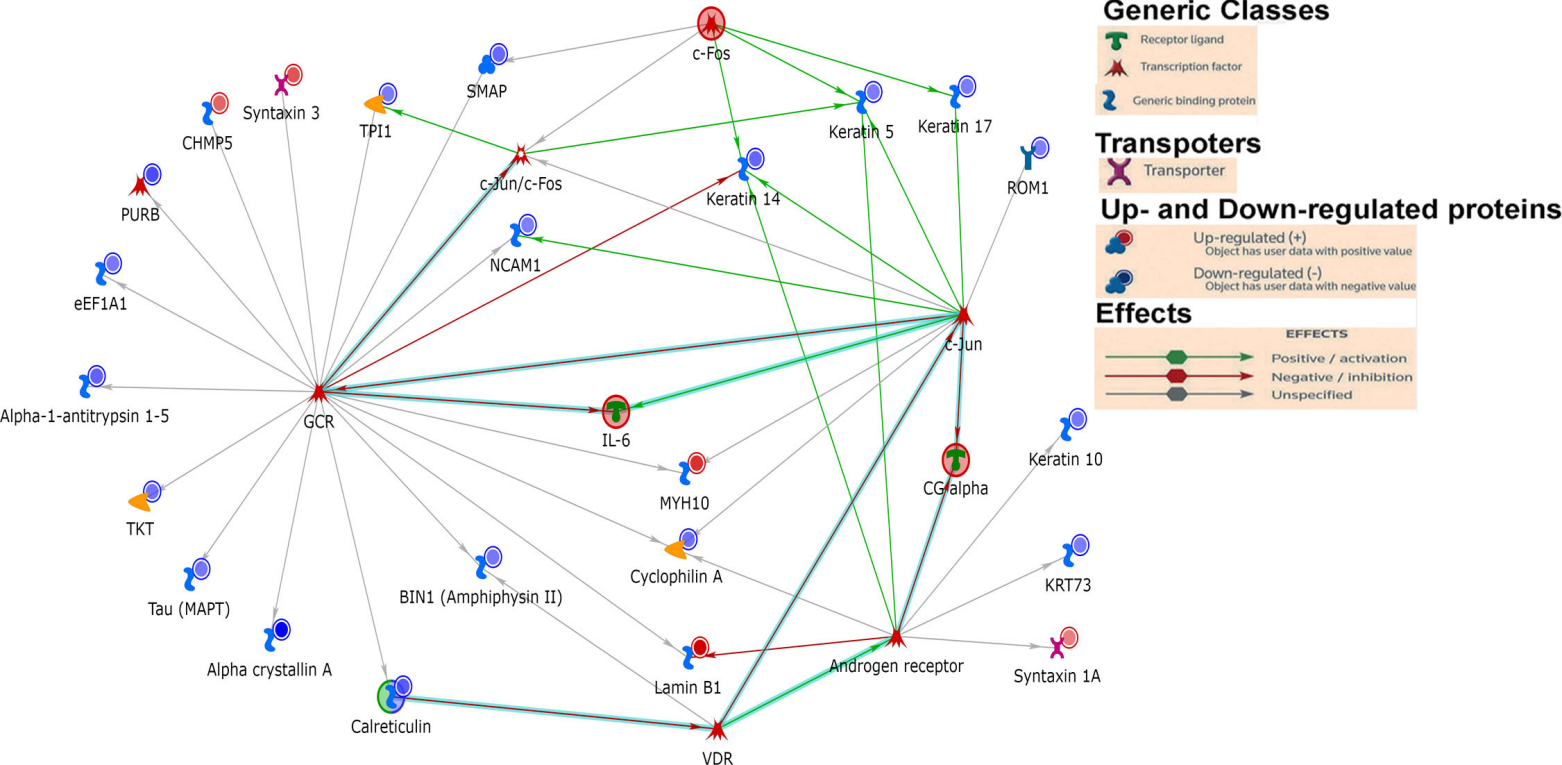
The network of proteins with elevated or reduced *O*-glycosylation in diabetic retinas.

First, we analyzed the level of *O*-GlcNAc-modified proteins by western blot analysis and learned that *O*-GlcNAcylation is increased in diabetic retina (**Figure 2B**). Because our LC-MS technique does not distinguish the different types of *O-*glycosylation, we next refer to proteins identified by this technique as *O*-linked glycosylated proteins. We again used an unbiased proteomics approach utilizing LC/MS to identity glycosylated peptides in both groups and compared the results (**Figures 4**, **5**, **Table 3**). We identified significant changes in *O*-glycosylation of peptides corresponding to 37 proteins. As expected, this is much fewer than detected in our global dataset. We identified differentially *O*-glycosylated proteins involved in metabolism, neuronal structure, and presynaptic vesicle docking. For example, diabetic retinas displayed reduced *O*-glycosylation of Triosephosphate isomerase (Tpi), Elongation factor 1-alpha 1(Eef1a1), and Transketolase demonstrating a reduction of *O*-glycosylation of proteins representing an array of metabolic processes ranging from glycolysis to protein synthesis. On the contrary, we found that *O-*glycosylation of Microfibrillar-associated protein 1 (Mfap1), a protein involved in Pre-mRNA splicing, and Charged multivesicular body protein 5 (Chmp5), a protein seemingly involved in a plethora of cellular processes including endosomal sorting, was elevated (29).

**Table 3:** Proteins with altered *O*-glycosylation in the diabetic retina are shown.

UniProtKB ID	Accession#	Fold(D/C)	Known *O*-Glycosylation Type & Sites	UniProtKB ID	Accession#	Fold(D/C)	Known *O*-Glycosylated Types & Sites
Lamin-B1	P14733	4.5	*O*-GlcNAc, Thr77	Keratin, type II cytoskeletal 5	Q922U2	-1.7	*O*-GlcNAc, Ser548
Myosin-10	Q61879	3.3	Not Applicable	Krt2 protein	B2RTP7	-1.7	Not Applicable
Syntaxin-3	Q64704	2.5	Not Applicable	Microtubule-associated protein tau	P10637	-1.8	*O*-GlcNAc, 4 sites
Rootletin	Q8CJ40	2.4	Not Applicable	Elongation factor 1-alpha 1	P10126	-1.8	*O*-GlcNAc
Microfibrillar-associated protein 1	Q9CQU1	2.3	Not Applicable	Myc box-dependent-interacting protein 1	O08539	-1.8	Not Applicable
Protein Lcn11	A2BHR2	2.3	Not Applicable	Keratin, type I cytoskeletal 10	P02535	-1.8	*O*-GlcNAc
Complexin-4	Q80WM3	2.2	Not Applicable	Transketolase	P40142	-1.9	*O*-GlcNAc
Charged multivesicular body protein 5	Q9D7S9	2.1	Not Applicable	Peptidyl-prolyl cis-trans isomerase A	P17742	-2.0	*O*-GlcNAc, Ser159
Soluble lamin-associated protein of 75 kDa	Q5XG69	1.7	Not Applicable	Hepatoma-derived growth factor-related protein 3	Q9JMG7	-2.1	Not Applicable
Syntaxin-1A	O35526	1.5	*O*-GlcNAc	Small acidic protein	Q9R0P4	-2.2	*O*-GlcNAc
Keratin Kb40	Q6IFT3	-1.5	Not Applicable	Keratin, type I cytoskeletal 14	Q61781	-2.3	Not Applicable
Rod outer segment membrane protein 1	P32958	-1.6	Not Applicable	Alpha-1-antitrypsin 1-5	Q00898	-2.4	Not Applicable
Heterogeneous nuclear ribonucleoprotein K	Q8BT23	-1.6	*O*-GlcNAc	Calreticulin	P14211	-2.6	*O*-GlcNAc
Neural cell adhesion molecule 1	P13595	-1.6	*O*-GlycNAc, 17 sites	Transcriptional activator protein Pur-beta	O35295	-3.0	*O*-GlcNAc
Triosephosphate isomerase	P17751	-1.7	*O*-GlcNAc	Keratin 16	Q3ZAW8	-3.2	Not Applicable
Keratin, type II cytoskeletal 73	Q6NXH9	-1.7	Not Applicable	Keratin, type I cytoskeletal 42	Q6IFX2	-3.6	*O*-GlcNAc
Keratin, type I cytoskeletal 17	Q9QWL7	-1.7	*O*-GlcNAc	Alpha-crystallin A chain	P24622	-5.2	*O*-GlcNAc, Ser185

To our surprise, we found elevated *O*-glycosylation of Syntaxin-1A, Syntaxin-3, and Complexin-4, proteins involved in synaptic vesicle docking and fusion (30, 31), in diabetic retinas. Syntaxin-1A is involved in neurotransmitter dependent endocytosis and exocytosis and part of the Soluble NSF Attachment Receptor (SNARE) complex (32). Syntaxin-3 is reportedly involved in the docking of synaptic vesicles (33, 34). Syntaxin-3 is essential for photoreceptor survival (35, 36), so elevated *O*-glycosylation of this protein in DR is certainly intriguing. *O*-glycosylation of Rootletin was also increased in the retinas of diabetic mice. Rootletin is an essential component of the ciliary rootlet (37, 38), which is present at the base of the primary cilium including those of photoreceptor outer segments (OS) (39).

We also detected reduced *O*-glycosylation of Neural cell adhesion molecule 1(Ncam1), Rod outer segment membrane protein 1 (Rom1) and Microtubule-associated protein tau. As its name suggests, Ncam1 is involved in neuron-neuron adhesion but also participates in the outgrowth of neurites (40). Rom1 is a protein vital to a range of processes in the rod photoreceptor OS including organization and disk maintenance. Of note, ROM1 mutations have been associated with retinitis pigmentosa (41). Another example is Tau, while best known for its association with neurodegenerative diseases (42), is also involved in various neuronal processes such as establishing neuronal polarity, structurally connecting components to the plasma membrane, and cytoskeletal stability (42). Reduced *O*-glycosylated pattern of Tau has been reported in postmortem brain of Alzheimer patients (43). It has been proposed that the glycosylation is responsible for its hyperphosphorylation and aggregation (44). Though it is unclear what *O*-glycosylation of these proteins regulates in the retina, it is interesting to see a reduction of these proteins as global levels of proteins with similar neuronal involvement was also observed.

## Discussion

Here we reported changes in the retinal proteome of mice with diabetes. We highlighted alterations in global proteins involved in metabolic processes, maintaining cellular structure, trafficking, and neuronal processes. We then showed alterations in the *O*-linked glycosylation of individual proteins in the diabetic retina.

We learn that biological processes such as UTP/GTP biosynthesis and cellular iron homeostasis were markedly enriched in diabetic retinas while negative regulation of p38/MAPK cascade was the most downregulated pathway. The alterations of metabolic pathways in the diabetic retina consist of major changes in the carbohydrate metabolism including TCA and glycolysis. These changes are usually associated with T1D and therefore, there is no wonder that STZ-induced diabetic mice manifest these alterations. Although presented by a smaller portion, the alterations in signaling pathways such as lipid metabolism detected in the diabetic retina of mice with T1D are primarily associated with T2D overall indicating that changes in fatty acid β-oxidation and TAG pathways could also be identified in the STZ-induce diabetes.

Diabetes is a complex systemic metabolic disorder with an interplay between several metabolic pathways in different organs of the human body. The diabetic retina is not an exclusion and together with neuropathy, nephropathy, and cardiopathy presents a complication of diabetes. Dyslipidemia involving both central as well as the local retinal specific mechanisms plays a critical role in the development of DR. Proteins responsible for fatty acid β-oxidation, phosphatidylcholine singling, triacylglycerol metabolism, and ceramide biogenesis are top-modified proteins in the retina of mice with 3 months of diabetes. The ABC transporter subunit A8 is one of them. Over 12-fold increase of these proteins is found in diabetic retinas (**Table 1**). ABCA8 as well as ABCA1 facilitate the efflux of cholesterol to lipid-free ApoA-I (45) and is proposed to participate in sphingomyelin (SM) production (46). SM is predominantly stored in membrane lipid rafts and significant increase in its level could impact membrane rigidity and permeability. Excess SM could also be converted into ceramide by the catalytic action of sphingomyelinase. This metabolic pathway is altered in diabetic retinas (**Figure 2**). Both altered sphingolipid and ceramide metabolism leading to inflammation and apoptotic cell death in diabetic retinas have been reported earlier (47, 48).

Another example is oxysterol binding protein 1 (OSBP1) which participates in lipid metabolism, regulation of secretory vesicle generation and signal transduction, and acts as a sterol sensor controlling a variety of sterol-dependent cellular processes. The level of this protein drops over 3-fold in the diabetic retina. Altogether these results suggest that lipid metabolism is dysregulated in the diabetic retina.

Recent studies on *O*-glycosylation revealed an abnormal protein PTM in varied human and mouse diabetic tissues(49–51). Moreover, in mice with T1D and T2D, the enhancement of protein *O*-glycosylation has been also reported (52–54) which is in agreement with our findings demonstrating increase in *O*-GlcNac-modified proteins (**Figure 2**). Despite the importance of the mentioned above studies and the present information on individual retinal cell types manifesting enhanced *O*-glycosylation, these studies, unfortunately, did not provide a list of individual proteins subjected to aberrant *O*-glycosylation. Therefore, to the best our knowledge, this is the first comprehensive study on the *O*-glycosylation of individual retinal proteins in early diabetes.

Knowing to be associated with the access of glucose, the *O*-GlcNAc modification is a dynamic process occurring in response to environmental stimuli. Two enzymes *O*-GlcNAc transferase (OGT) and *O*-GlcNAcase (OGA) participate in dynamic *O*-glycosylation. OGT catalyzes the addition of the *O*-GlcNAc moiety while the OGA is responsible for its removal. Surprisingly, while the OGA level was not changed in diabetic retinas, the production of nuclear OGT1 (110 kD) was reduced by 30% as compared to the control (**Supplementary Table S1**). Although this reduction is in agreement with a reduction in mTOR, S6K, and eIF4G proteins known to be responsible for mRNA translation (**Figure 1** and **Table 1**) and coincide with the data on selected and overall reduced mRNA translation reported in a hyperglycemic environment (55), our results and the reported studies indicated an increase in *O*-GlcNAc proteins overall (**Figure 2** and (52–54). One of the potential explanations for this phenomenon is that STZ could inhibit OGA (56) and the reduction in OGT is a compensatory feedback to this inhibition. It is possible that the OGT/OGA ratio is highly dynamic, and we caught the changes in OGT and did not detect ones in OGA. Nevertheless, like the study with Akita mice, future experiments should identify an OGT1/OGA ratio in the retina of STZ-induced mice at different stages of DR progression, since the abnormal cycling has been reported to induce early cell death of retinal pericytes, one of the earliest signs of DR (52, 57).

The *O*-glycosylation could occur either in the cytoplasm or the nucleus. In our study, nuclear Lamin B1 demonstrates the highest hit for the modified *O*-glycosylated proteins in the diabetic retina. It has been reported that Lamins (A, B, and C) have the highest *O*-glycosylation levels among all nuclear proteins in the cell (58). Therefore, it is possible that the threshold for *O*-Glycosylation detection used in the study was high and this led us to limited numbers of over-glycosylated proteins overall (**Table 3**). Despite this potential limitation, we observed an increase in the *O*-glycosylation pattern of rootletin. Rootletin is known to localize at the basal body of photoreceptors and extend to its synaptic terminal. Little is known about PTMs of this protein in healthy and in diseased retinas. Besides the knowledge of 2 *O*-glycosylated sites (Ser1065 and Ser1892) located in rootletin (GlyGen, Q5TZA2-1), no study has been conducted to elucidate the role of *O*-Glycosylation in this protein. Therefore, future studies should reveal the role of rootletin *O*-glycosylation in photoreceptor homeostasis.

Another discovery of our study was the enhanced *O*-glycosylation of Syntaxins (1A and 3) in the diabetic retina. While the role of *O*-glycosylation in the syntaxin activity has not been explored, the study by Delacour et al. has demonstrated that treatment of polarized HT-29 cells with 1-benzyl-2-acetamido-2-deoxy-D-galactopyranoside (GalNAc-*O*-bn), an inhibitor of glycosyltransferase incorporating glucosamine into *O*-glycans, induce a shift into intracellular distribution of Syntaxin 3 vs apical membrane localization (59). The study proposed that the aberrant *O*-glycosylation may affect Syntaxins cellular localization. Of note, the expression of Syntaxin 1A is reduced in the T1D (STZ-induced) Wistar rat and T2D db/db mouse retinas (60, 61).

Calreticulin demonstrates reduced *O*-glycosylated pattern in diabetic retinas as compared to control. Although glycosylation of calreticulin has been shown in the liver and brain, it has been also reported to be lacking in other tissues including human lymphocytes (62). Here we reported that the retinal tissue also manifests calreticulin *O*-glycosylation similar to the brain. However, the certain type of *O*-Glycosylation and the potential role this PTM could play in varied tissues and cells have not been revealed. Recently, a study conducted with tunicamycin-induced inhibition of glycosylation has proposed that aberrant glycosylation enhances pro-inflammatory activity and immunogenicity without affecting the monomeric status of calreticulin (63). For example, treatment of mouse peritoneal macrophages with recombinant calreticulin manifesting reduced *O*-glycosylation results in elevated secretion of pro-inflammatory cytokines TNFa and Il-6. Of note, inflammation is known to be a hallmark of diabetes that promotes the development of diabetic retinopathy in patients. Therefore, it is possible, that the observed 2.6-fold reduction in calreticulin glycosylation contributes to the immune response in the diabetic retina.

Another example of the reduced *O*-glycosylate pattern in our study was alpha-1-antitrypsin (A1AT). Although this protein is mainly produced by hepatocytes, it could also derive from macrophages, monocytes, and other cells. Thus, the retinas of C57BL6 mice showed A1AT expression mainly distributed in the inner nuclear layer and is mostly co-localized with CD68 and CD11b positive staining, markers of microglia in the retina (64). Knowing to be a therapeutic target for the treatment of chronic obstructive pulmonary disease, recently A1AT has been proposed as a candidate target to treat diabetic retinopathy due to its involvement in anti-inflammatory processes, anti-apoptotic activity, extracellular matrix remodeling, and protection of vessel walls and capillaries via NOS-mediated NO suppression (65, 66) The study by Ortiz and colleagues demonstrated that treatment of diabetic mice with A1AT results in reduced inflammation and retinal degeneration (67). Consequently, more information on the mechanism of A1AT action in diabetic retinas should be gained including glycosylation as a PTM.

The A1AT molecule is composed of 394 amino acids and is posttranslationally modified by N-glycosidically linked oligosaccharides at three asparagine residues at positions 70, 107, and 271 (67). Several glycoforms of A1AT exist which are assigned different properties regarding half-life, stability, and the immune modulatory properties of A1AT (68). Because it is well studied, N-glycosylation is a primary focus of A1AT PTM research. It is known that recombinant A1AT with an extended half-life and protection from agglomeration is differently glycosylated than human-purified A1AT which is prone to polymerization. Therefore, glycosylation is needed for A1AT secretion. In addition, A1AT plays a multifaceted role in inflammation by affecting the expression of TNFα, IL-6, IL-8, nFN-γ, and IL-1-β. The study demonstrated that non-glycosylated recombinant A1AT does not possess the same anti-inflammatory effect as glycosylated human-purified A1AT. Interestingly, both A1AT expression and *O*-glycosylation were significantly reduced in our study. While low A1AT expression (25-fold decrease) is in agreement with the observed diminishing A1AT in rd1 mice with severe retinopathy (64), the role of reduced *O*-glycosylation (over 2-fold decrease) needs to be explored further in detail.

In the diabetic retina, alpha Crystallin A (Cryaa) manifests the lowest level of *O*-glycosylation (over 5-fold as compared to control). Cryaa is a small heat shock protein acting as a molecular chaperone holding misfolded proteins in large soluble aggregates. It has been originally proposed that Cryaa expression is mostly restricted to the lens in the human body. Later, Deretic and colleagues reported that alpha A- and alpha B-Crystallins bound specifically to the photoreceptor post-Golgi membranes to mediate the transport of newly synthesized rhodopsin in frog retinal photoreceptors (69). The studies reported that Cryaa serves as a protective factor in retinal pathological processes and is strongly elevated after injury and light damage (70, 71).

While the mechanism of Cryaa-mediated protection is still under investigation, the Cryaa chaperoning activity could be associated with its PTM. For example, nonenzymatic glycation of Cryaa is 2-fold higher in the diabetic lens than in the normal lenses (72). It has been proposed that increased glycation correlates with an increase in the size of aggregates formed by Cryaa leading to a decrease in chaperoning activity (73). Altogether, these data suggest that Cryaa loses its cytoprotective during diabetes. Oppositely, metabolic product methylglyoxal binds and post-translationally enhances the Cryaa activity (74). These data suggest that the functional role of Cryaa PMTs should be carefully examined. For example, the functional role of *O*-GlcNac in Cryaa is unknown. Attachment of *O*-GlcNac occurring at serine 162 has been proposed to increase the resistant of Cryaa to PNGase F cleavage (75). Therefore, the observed reduction in *O*-Glycosylation in Cryaa in the diabetic retina could be associated with an increase in Cryaa agglomeration and/or solubility leading to the loss of its protective effect. Additionally, rapid phosphorylation prolonging its half-life could also be a reason as well. Notably, cone opsins, main retinal proteins, did not manifest changes in the *O*-glycosylation pattern in diabetes; both *O*- and N-glycosylation occur on these proteins at the N-terminal (76).

## Conclusion

We have identified top-rated proteins with altered O-glycosylation in the diabetic retina. One of the limitations of the current study is that it doesn’t not distinguish between different types of *O*-Glycosylation. A search of the literature demonstrated that little is known about the posttranslational *O*-glycosylation of these proteins in the diabetic retina, for example *O*-GlcNAcylation or *O*-GalNAcylation. In addition to the discovery of new sites within proteins for example, *O*-GalNAcylation and *O*-GlcNAcylation, future studies should provide information on the role of individual protein *O*-Glycosylation to understand the consequences of altered PTM in diabetic retinas. Not only these studies are critical to enhancing our knowledge of protein structure, function, and localization but also they ensure the future development of therapeutic strategies targeting individual protein PTMomes in the diabetic retina.

## Materials and methods

### Animals

All animal procedures were approved by The University of Alabama at Birmingham institutional animal use and care (IACUC) committee and in accordance with the statement for the Use of Animals in Ophthalmic and Vision Research by The Association for Research in Vision and Ophthalmology (ARVO). C57BL/6J mice were purchased from Jackson Laboratory (Bar Harbor, ME). Diabetes was induced in mice as described previously. Briefly, 2 month-old-C57BL6 males were subjected to daily injections of 50 mg/kg STZ (streptozotocin) or vehicle (0.1mol/L citrate buffer,pH4.5) during 5 consecutive days. Hyperglycemia in mice was recorded by measuring blood glycose levels (Hb1Ac) as previously described (5).

### Proteomics analysis

#### Sample preparation

Proteomics analysis was carried out as previously referenced with minor changes [(77), within section 2.5 nLC-ESI-MS2 under Protein IDs for GeLC]. Mice were euthanized by CO2 asphyxiation, then retinas were harvested and proteins were extracted using T-PER™ Mammalian Protein Extraction Reagent (Thermo Fisher Scientific, Cat.# 78510) supplemented with HALT protease inhibitor cocktail (Thermo Fisher Scientific, Cat.# 78425), and benzonase nuclease (Sigma, E1014) following manufacturers instructions. Lysates were quantified using Pierce BCA Protein Assay Kit (Thermo Fisher Scientific, Cat.# 23227). Protein extracts were divided to carry out both “global” and “*O*-glycan enriched” proteomics analysis, whereby the later preparation included overnight digestion of 300ug of protein with PNGase-F (Sigma, P7367) as per manufacturers recommendation. To detect *O*-glycosylation, the PNGase-F treated lysates were then processed to enrich *O*-linked glycosylated protein using a ConA glycoprotein isolation kit (Thermo Fisher Scientific, Cat.# 89804). ConA is known to be useful for the separating *O*-Glycosylated from N-Glycosylation proteins removed by PNGase-F treatment in the first place. Therefore, the ConA bound fraction was released and analyzed further as per the global proteome workflow. For global analysis 20ug of protein was separated half way on the gel, and for the *O*-glycan enriched proteins 1ug was run as a short stack into gel. In both cases, protein per sample was diluted to 25µL using NuPAGE LDS sample buffer (1x final conc., Invitrogen, Cat.# NP0007). Proteins were then reduced with DTT and denatured at 70°C for 10min prior to loading everything onto Novex NuPAGE 10% Bis-Tris Protein gels (Invitrogen, Cat.# NP0315BOX) and separated appropriately (@ 200 constant V). The gels were stained overnight with Novex Colloidal Blue Staining kit (Invitrogen, Cat.# LC6025). Following de-staining, each entire lane was cut into multiple MW fractions (1-fraction for the *O*-glycan enriched, and 3 fractions for the global), and equilibrated in 100 mM ammonium bicarbonate (AmBc), each gel plug was then digested overnight with Trypsin Gold, Mass Spectrometry Grade (Promega, Cat.# V5280) following manufacturer’s instruction. Peptide extracts were reconstituted in 0.1% Formic Acid/ddH_2_O at 0.1µg/µL.

#### Mass spectrometry

Peptide digests (8µL each) were injected onto a 1260 Infinity nHPLC stack (Agilent Technologies), and separated using a 75 micron I.D. x 15 cm pulled tip C-18 column (Jupiter C-18 300 Å, 5 micron, Phenomenex). This system runs in-line with a Thermo Q Exactive HFx mass spectrometer, equipped with a Nanospray Flex™ ion source (Thermo Fisher Scientific), and all data were collected in CID mode. The nHPLC is configured with binary mobile phases that includes solvent A (0.1%FA in ddH_2_O), and solvent B (0.1%FA in 15% ddH_2_O/85% ACN), programmed as follows; 10min @ 5%B (2µL/min, load), 30min @ 5%-40%B (linear: 0.5nL/min, analyze), 5min @ 70%B (2µL/min, wash), 10min @ 0%B (2µL/min, equilibrate). Following each parent ion scan (300-1200m/z @ 60k resolution), fragmentation data (MS2) were collected on the top most intense 18 ions @7.5K resolution. For data dependent scans, charge state screening and dynamic exclusion were enabled with a repeat count of 2, repeat duration of 30s, and exclusion duration of 90s.

#### MS data conversion and searches

The XCalibur RAW files were collected in profile mode, centroided and converted to MzXML using ReAdW v. 3.5.1. The mgf files were created using MzXML2Search (included in TPP v. 3.5) for all scans. The data was searched using SEQUEST (Thermo Fisher Scientific), which is set for three maximum missed cleavages, a precursor mass window of 20ppm, trypsin digestion, variable modification C @ 57.0293, and M @ 15.9949 as a base setting. Searches were performed with the mus musculus species specific subset of the UniProtKB database.

#### Peptide filtering, grouping, and quantification

The list of peptide IDs generated based on SEQUEST search results were filtered using Scaffold (Protein Sciences, Portland Oregon). Scaffold filters and groups all peptides to generate and retain only high confidence IDs while also generating normalized spectral counts (N-SC’s) across all samples for the purpose of relative quantification. The filter cut-off values were set with minimum peptide length of >5 AA’s, with no MH+1 charge states, with peptide probabilities of >80% C.I., and with the number of peptides per protein ≥2. The protein probabilities will be set to a >99.0% C.I., and an FDR<1.0. Scaffold incorporates the two most common methods for statistical validation of large proteome datasets, the false discovery rate (FDR) and protein probability (Keller, Nesvizhskii, Weatherly). Relative quantification across experiments were then performed via spectral counting (Old, Liu), and when relevant, spectral count abundances will then be normalized between samples (Hyde).

#### Statistical analysis

For the proteomic data generated, two separate non-parametric-like statistical analyses were performed between each pair-wise comparison. These analyses included; 1) the calculation of weight values by significance analysis of microarray (SAM; cut off >|0.8| combined with, 2) T-Test (single tail, unequal variance, cut off of p < 0.05), which are then sorted according to the highest statistical relevance in each comparison. For SAM (Golub and Xu), whereby the weight value (W) is a statistically derived function that approaches significance as the distance between the means (μ1-μ2) for each group increases, and the SD (δ1-δ2) decreases using the formula, W=(μ1-μ2)/(δ1-δ2). For protein abundance ratios determined with N-SC’s, we set a 1.5-fold change as the threshold for significance, determined empirically by analyzing the inner-quartile data from the control experiments using ln-ln plots, where the Pierson’s correlation coefficient (R) is 0.98, and >99% of the normalized intensities fell between the set fold change. In each case, all three tests (SAM, Ttest, and fold change) have to pass in order to be considered significant.

*Systems Analysis:* Gene ontology assignments and pathway analysis will be carried out using MetaCore (GeneGO Inc., St. Joseph, MI) and ShinyGo (78). Interactions identified within MetaCore are manually correlated using full text articles. Detailed algorithms have been described previously [Bhatia and Ekins]. doi: 10.1093/bioinformatics/btz931.

### Immunoblotting

Retinas from control and STZ induced diabetic animals were dissected and lysed with RIPA buffer (Cell signaling, Cat#9806) supplemented with Halt protease and phosphatase inhibitor cocktail following the manufacturer’s instructions. Homogenized retina extracts rotated for 30 min at 4 °C, centrifuged 12000g for 10min at 4 °C and the supernatant was collected for protein estimation (BioRad Cat#5000001). 70 μg of protein was separated by SDS-PAGE and transferred to a PVDF membrane for immunoblotting. Primary antibodies were obtained from Cell Signaling Technology (p70-S6K;Cat#2708T, *O*-GlcNac (CDT110,6;Cat#9875) or from Millipore-Sigma (beta actin; Cat#2066). HRP- conjugated secondary antibodies were obtained from LI-COR (HRP Goat anti-Mouse IgG;Cat#926-80010, HRP Goat ant-Rabbit;Cat#926-80011).

## Data availability statement

The global mass spectrometry proteomics data presented in this study have been deposited to the ProteomeXchange Consortium via the PRIDE partner repository, accession number PXD044269. The mass spectrometry proteomics data pertaining to the glycoproteomics presented in this study have been deposited to the ProteomeXchange Consortium via the PRIDE partner repository, accession number PXD044270.

## Ethics statement

The animal study was reviewed and approved by UAB IACUC committee.

## Author contributions

CS, JM, and MG analyzed data, wrote, edited, and approved the manuscript, MG designed experiments. CS, JM, AZ, and MG prepared figures. CS and AZ conducted experiments. All authors contributed to the article and approved the submitted version.

## Supplementary material

Supplementary Table 1 Global proteins modified in diabetic retinas.
